# Low-Cost Dielectric Substrate for Designing Low Profile Multiband Monopole Microstrip Antenna

**DOI:** 10.1155/2014/183741

**Published:** 2014-07-20

**Authors:** M. R. Ahsan, M. T. Islam, M. Habib Ullah, H. Arshad, M. F. Mansor

**Affiliations:** ^1^Department of Electrical, Electronic and Systems Engineering, Faculty of Engineering and Built Environment, Universiti Kebangsaan Malaysia (UKM), 43600 Bangi, Selangor, Malaysia; ^2^School of Information Technology, Faculty of Information Science and Technology, Universiti Kebangsaan Malaysia (UKM), 43600 Bangi, Selangor, Malaysia

## Abstract

This paper proposes a small sized, low-cost multiband monopole antenna which can cover the WiMAX bands and C-band. The proposed antenna of 20 × 20 mm^2^ radiating patch is printed on cost effective 1.6 mm thick fiberglass polymer resin dielectric material substrate and fed by 4 mm long microstrip line. The finite element method based, full wave electromagnetic simulator HFSS is efficiently utilized for designing and analyzing the proposed antenna and the antenna parameters are measured in a standard far-field anechoic chamber. The experimental results show that the prototype of the antenna has achieved operating bandwidths (voltage stand wave ratio (VSWR) less than 2) 360 MHz (2.53–2.89 GHz) and 440 MHz (3.47–3.91 GHz) for WiMAX and 1550 MHz (6.28–7.83 GHz) for C-band. The simulated and measured results for VSWR, radiation patterns, and gain are well matched. Nearly omnidirectional radiation patterns are achieved and the peak gains are of 3.62 dBi, 3.67 dBi, and 5.7 dBi at 2.66 GHz, 3.65 GHz, and 6.58 GHz, respectively.

## 1. Introduction

The modern technological advancement and emerging trends in the area of wireless communications raise considerable research interest in antenna designs to integrate easily with system by ensuring the low physical profile with multifunctionality in a single device. Moreover, the recent improvement and versatile use of personal communications and portable devices necessitate the mandatory use of low-cost, lightweight, compact, and multifrequency antenna. Printed microstrip patch antennas are competitive solutions for their inherent advantages of low-cost, low-profile, lightweight, less troublesome fabrication, and ease of integration to the system [1, 2]. In general, the antenna performance and its dimension are essentially interlinked together; an antenna performance is said to be good when its resonance and size are analogous to the wavelength. To deal with the current and future mobile communications, wireless services, and satellite applications, a multiband/multifunctional microstrip patch antenna associated with high performance and good radiation characteristics is certainly required [3–5]. An extensive research efforts have been contributed by the researchers in the augmentation of patch antenna performances by integrating various technologies to make it small and operate in several discrete frequencies while ensuring overall steady performance. Despite design complexities associated with multiband antenna, many researchers have discussed the design configuration of patch antenna which can operate more than one frequency band. A number of studies accompanying the applications and techniques have been reported in designing multiband antennas, making tapered structure with coplanar waveguide (CPW) fed [6, 7], configuring slots over the radiating patch [8, 9], introducing capacitive coupled patch [10, 11], employing multilayered structure [12–14], integrating electromagnetic band gap (EBG) structure [15, 16], and proposing metamaterials [17, 18]. In recent times, the Worldwide interoperability for Microwave Access (WiMAX) operating at 2.5/3.5/5.5 GHz bands is becoming very popular due to its strategic features [6, 19]. Whereas microwave C-bands operating at 4 to 8 GHz have advantages over the Ku-band and the developing regions in Africa, Asia sees a promising expansion of C-band satellite applications in near future [20–22].

Through reviewing a number of articles it has been found that numerous studies have been performed in the design of multiband patch antenna. A double G-shaped planar multiband antenna of 40 × 30 mm^2^ has been designed for WLAN, WiMAX, and HIPERLAN2 [23]. A 50 × 50 mm^2^ slot ring antenna integrated with capacitive patch has been proposed which is able to function at frequencies related to WLAN and WiMAX applications [10]. Coplanar waveguide- (CPW-) fed slotted patch antennas of 23 × 30 mm^2^ to operate in 2.4–2.63, 3.23–3.8, and 5.15–5.98 GHz bands [24] and 25 × 25 mm^2^ to cover 2.14–2.85, 3.29–4.08, and 5.02–6.09 GHz bands [6] have been developed. Two-U slot shaped patch antenna of 40 × 50 mm^2^ with three resonant frequencies 2.7, 3.3, and 5.3 GHz has been implemented to cover the triband wireless system [25]. In all aforementioned proposals and designs, the main target of the researchers is to achieve multiband antenna by compromising either fabrication cost or effective electrical area of the patch or steady radiation performance or gain or efficiency. In spite of everything, opportunities are ahead to research for designing a low profile patch antenna with high gain, good radiation characteristics, and high efficiency.

Based on the background of the researches above, this paper proposes a simple and small form factor multifunctional patch antenna fabricated at low cost, durable fiberglass polymer resin material substrate. The consumer markets for microwave applications and circuitry are gradually expanding with contemporary technological advancement; a substantial interest is always there in reducing cost for the system. The fibre glass polymer resin (FR4) substrates are very often to be chosen for RF and microwave circuitry due to their inexpensive market price in comparison with other available substrates. The material has dielectric constant 2.4; a typical loss tangent is 0.02 which is comparatively high for various printed circuit board applications. The dimensional stability is considerably good against temperature and frequency. Generally speaking, the fibre glass polymer resin substrate is not a good material for electrically large antennas and beamforming designs because of its high losses [26, 27]. On the contrary, for the RF and microwave applications where the losses and dielectric constants are less important, it can be used successfully by replacing other conventional expensive substrates. The proposed multiband patch antenna of 20 × 20 mm^2^ has been designed and fabricated, and its performance criteria have been critically analyzed by comparing other similar work.

## 2. Antenna Geometry and Design Process

**Figure 1:**
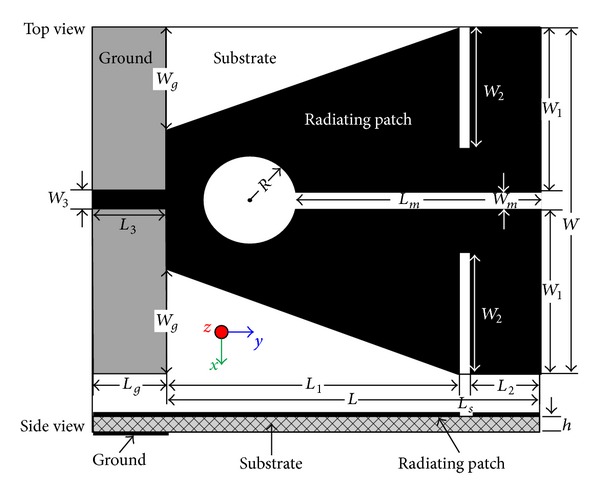
Geometry of the proposed multiband antenna.

The geometrical structure and configuration with detailed design parameters of the proposed patch antenna are shown in Figure 1. The antenna is designed and fabricated on an *h* = 1.6 mm thick fiberglass polymer resin substrate with relative dielectric constant *ε*_*r*_ = 4.6 and loss tangent tan*δ* = 0.02. The industry standard, 3D full wave electromagnetic field simulation tool HFSS package which is based on the finite element method (FEM) has been used for the design and simulation of the proposed antenna. The in house LPKF PCB prototyping machine is used in the prototype fabrication process. The two-sided structure of the antenna consists of a standard 50Ω SMA connector, a microstrip line for feeding, radiating patch on top, and a reduced rectangular ground plane. The proposed antenna structure is achieved by cutting slots and etching out different shapes from a conventional rectangular patch.

The analytical study shows that the width of the patch has insignificant effect on obtaining resonance and through mathematical modeling the patch width for desired frequency can be calculated by utilizing the already established mathematical equations [28]. The length *L* of the radiating patch has dominating effect on the antenna performances other than the width *W*. Consider

(1) $$\begin{array}{c} W=\frac{c}{2f_{o}}\sqrt{\frac{\varepsilon_{r}+1}{2}}, \end{array}$$

(2) $$\begin{array}{c} L=\frac{c}{2f_{o}\sqrt{\varepsilon_{r}}}-2\Delta L. \end{array}$$

The usual symbol *W* is the width and *L* is the length of the radiating patch in (1) and (2), respectively, whereas *c* is the speed of light, *f*_*o*_ is the center frequency, *ε*_*r*_ is the relative dielectric constant, and Δ*L* is the change in length. The effective dielectric constant, *ε*_eff_, can be formulated as

(3) $$\begin{array}{c} \varepsilon_{\text{eff}}=\frac{1}{2}\left(\varepsilon_{r}+1\right)+\frac{1}{2}\left(\varepsilon_{r}-1\right)\sqrt{\left(1+\frac{10h}{W}\right)}, \end{array}$$

where *h* denotes the thickness of the substrate used.

Because of the effect of the fringing field surrounding the radiating patch, the electrical dimension of the antenna seems to be bigger than the physical dimension. The change of length of Δ*L* due to the effect of fringing field can be presented by the following:

(4) $$\begin{array}{c} \Delta L=0.412h\frac{\left(\varepsilon_{e}+0.3\right)\left[\left(W/h\right)+0.264\right]}{\left(\varepsilon_{e}-0.258\right)\left[\left(W/h\right)+0.8\right]}. \end{array}$$

The available equations are applicable for conventional rectangular radiating patch; however the geometric shape and dimension of the proposed antenna have been achieved by modify, test, and run method. The dimension of the microstrip line is optimized through design and simulation to obtain enhanced impedance matching over the operating frequency bands. The designing process of the antenna is started with estimating that the overall dimension of radiating patch is responsible for providing the compact size of the antenna.

**Figure 2:**
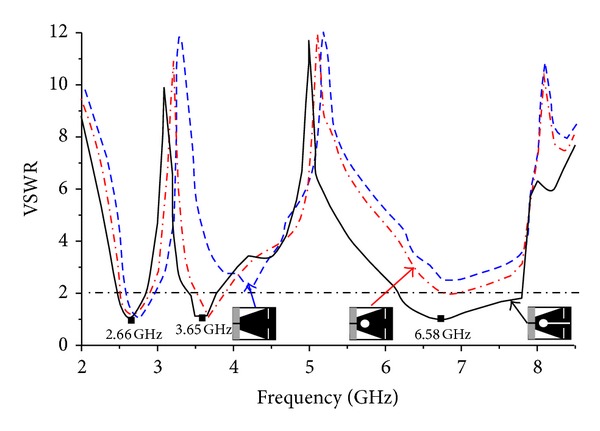
Simulated VSWR for different geometrical structure of radiating patch.

The evolution of the final radiating patch has been developed by etching some of the parts in a different shape from the conventional rectangular patch. Through numerous simulation analysis, it has already been verified that the placement of narrow strips and/or cutting slits is accountable in creating meandered path for the surface currents which mainly are responsible for producing the resonant frequencies. To examine the effects of introducing different slots embedded with the rectangular patch, investigations on VSWR and resonant frequencies have been performed which is illustrated in Figure 2.

**Figure 3:**
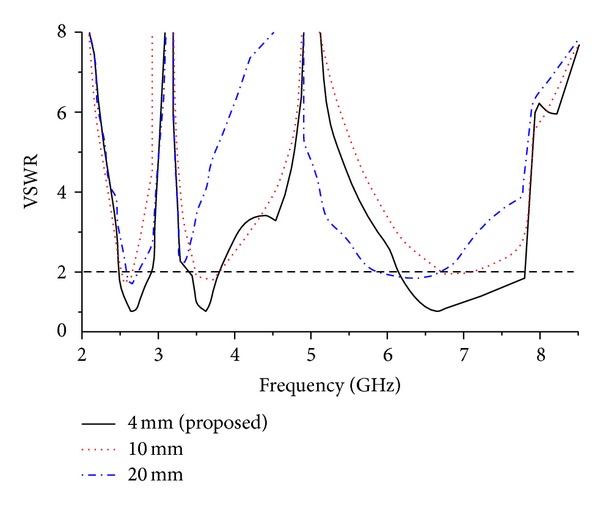
Effect of ground plane on the VSWR of the proposed antenna.

**Figure 4:**
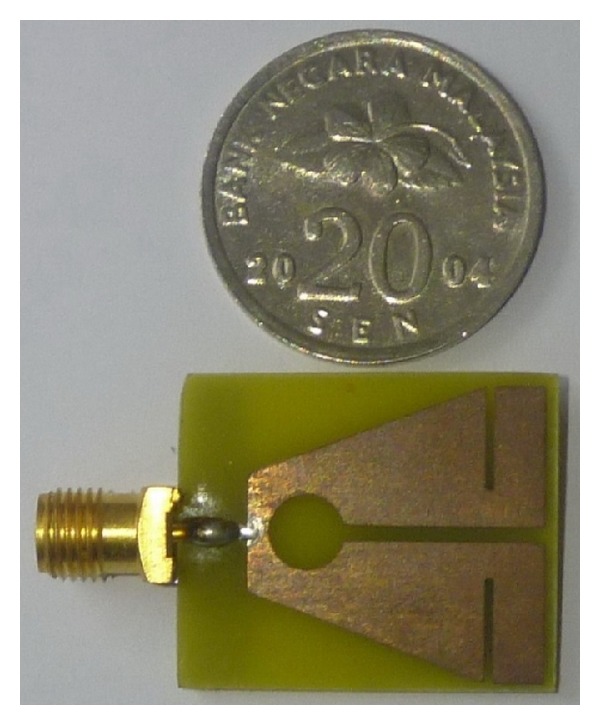
Prototype of the developed multiband antenna.

The combinational effect of triangular cut at the lower part of the patch and the pair of horizontal slits at the top produces a lower resonant frequency. This can be due to the increased flow of surface currents around the triangular cut sides and the top slits as clearly observed in the figures for surface currents (refer to surface current distribution in Figure 10(a)). Introducing the circle in the patch creates the second resonant frequency (at around 4.32 GHz) along with the lower resonant frequency. In this case, the first resonant frequency is shifted a bit and the VSWR also reduced as expected. Observing the surface current distribution of the second resonant frequency has clearly validated this in which situation the currents become more concentrated near the circular slot. Finally, the addition of middle slot with the triangular cut, top slits, and circle cut is accountable for generating the third resonant frequency. Not only does this insertion of middle slot affect the two lower frequency bands by shifting to desired resonant frequency and reducing the VSWR, but also their combined effect yields the wider band for higher resonant frequency. By examining the surface current distribution presented in Figure 10(c), it is seen that increased surface currents are converging near the middle slot. The effects of different ground plane dimensions have also been analyzed for the proposed antenna. According to the radiation principle of microstrip patch antenna, an equal and opposite direction current is created on the ground plane and in phase accumulation occurs with that of patch antenna. The length of the ground plane has a dominant effect on resonant frequency and impedance bandwidth [29–31].  Figure 3 shows three types of ground plane length on the VSWR against frequency. From the figure it can be concluded that the ground plane of 4 mm long and 20 mm wide gives a better VSWR performance with adequate bandwidth to cover WiMAX bands and C-band. After successful completion of the parametric studies in the simulation process, the optimal geometrical structure of the proposed antenna has been achieved depending on the expected frequency bands. The overall size of the patch is of 20 × 20 mm^2^ and the complete dimensions for optimized design parameters are given in Table 1. The prototype of the proposed antenna has been fabricated and is presented in Figure 4.

## 3. Result Analysis

**Figure 5:**
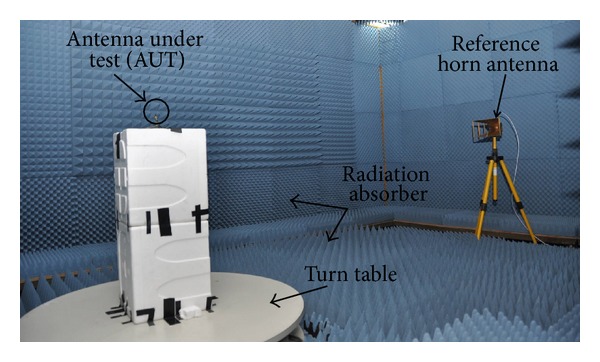
Photograph of the anechoic measurement chamber.

After successful completion of the design aspects, a prototype has been constructed and measured. With the aid of Agilent's vector network analyzer (VNA, Agilent E8362C), the antenna parameters have been measured in a standard far-field anechoic measurement chamber (5.5 × 4.5 × 4 m^3^). The photograph of the anechoic chamber is presented in Figure 5. The floor, roof, and wall of the chamber are covered with arrays of pyramid-shaped foam as a radiation absorbent material (less than −60 dB reflectivity). A turn table of 1.2 m diameter has been used to rotate the antenna under test (AUT) specimen at 1 RPM speed, which can cover 360 degrees. A 10-meter cable is used to connect the controller and VNA. A pyramidal horn antenna has been used as reference antenna and placed on top of the antenna sliding positioner. The simulated and measured outputs for antenna parameters have been further analyzed and graphically presented by available software package and computer aided tools.

**Figure 6:**
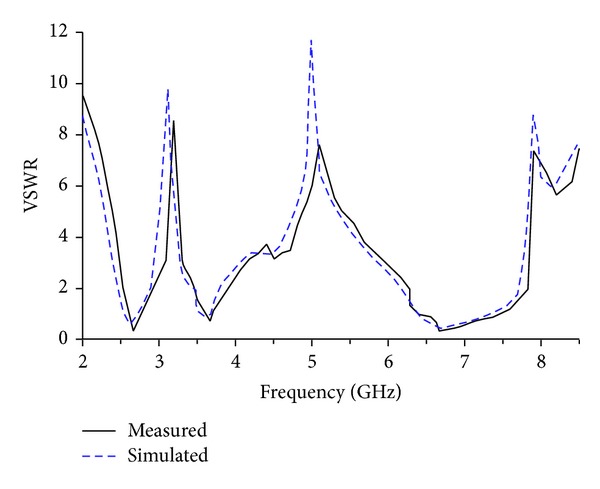
Measured and simulated VSWR of the proposed antenna.

The measured and simulated VSWR versus frequency of the proposed antenna has been presented in Figure 6. It is readily apparent from the figure that an excellent agreement has been attained between simulated and measured results. Three distinct impedance bandwidths with VSWR less than 2 are achieved: 360 MHz from 2.53 to 2.89 GHz, 440 MHz from 3.47 to 3.91 GHz, and 1550 MHz from 6.28 to 7.83 GHz, which are able to cover the 2.5/3.5 GHz WiMAX band and C-band, respectively. A little variation in between the simulated and measured result may be due to the fabrication tolerance, SMA soldering effect, and/or inconsistency in the dielectric constant of the material used.

**Figure 7:**
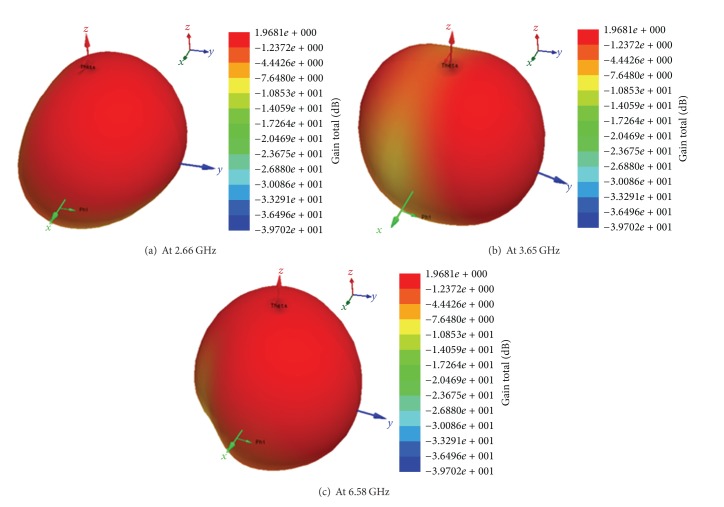
3D radiation pattern of the proposed antenna.

**Figure 8:**
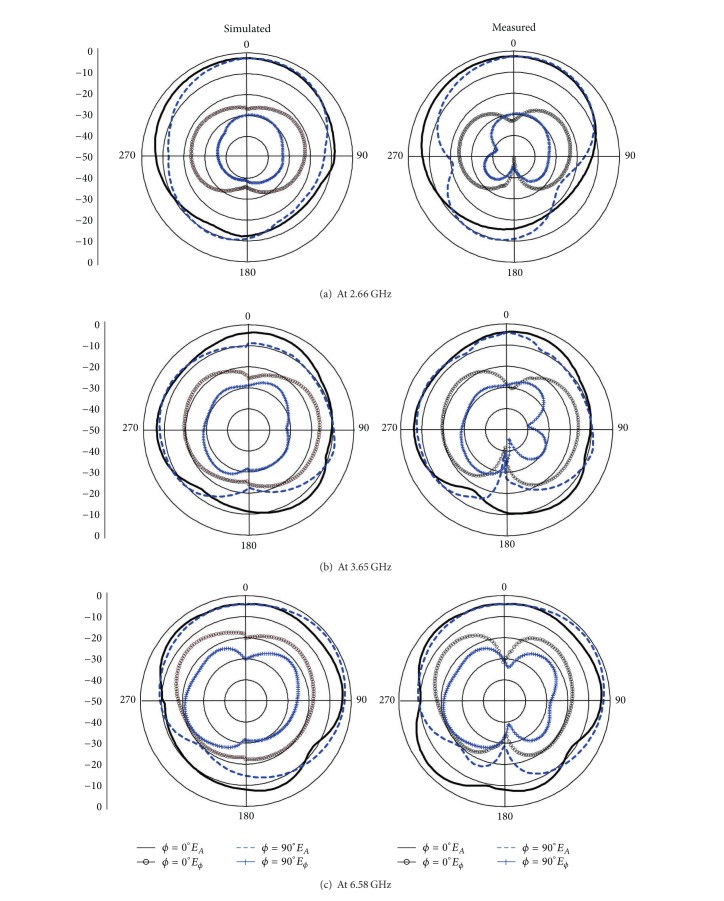
Simulated and measured radiation patterns of the proposed antenna.

3D polar plot of far-field radiation patterns for the proposed antenna is shown in Figure 7.  Figure 8 exhibits the comparison between the measured and simulated far-field radiation patterns in *E*-plane and *H*-plane for the frequencies at 2.66, 3.65, and 6.58 GHz. A little inconsistency can be spotted in measured results, more specifically the backward radiation. This may be due to the cable loss which is interposed between the antenna and controller. Other than this, the presented results indicate fairly good and steady patterns in the plane over the operating frequency bands. The copolarization patterns for *E*- and *H*-plane are almost symmetric and directional, whereas the cross polarization radiation patterns at different operating frequency bands, though, seem almost similar; however, their effect is observed to be increased with the increased frequencies as predicted. It has been observed that the designed antenna performs well in producing a nearly balanced radiation pattern radially for operating bands by maintaining low cross polarization as desired. These performance criteria would be certainly beneficial while designing antenna arrays and thus reasonably would produce a more stable radiation pattern across the operating frequency bands.

Free-space ranges are used to measure the gain of the designed multiband antenna. With the guidance from the IEEE standards [32] and following the well-known Friis transmission equation [33], the peak gain of the fabricated AUT is measured by utilizing two identical horn antennas whose gain and radiation patterns are known. The following equations are used for gain measurement.

 Antenna-1 (horn) and Antenna-2 (horn):
  (5) $$\begin{array}{c} G_{1\;\text{dB}}+G_{2\;\text{dB}}=20\;\text{lo}{\text{g}}_{10}\left(\frac{4\pi R}{\lambda }\right)+10\;\text{lo}{\text{g}}_{10}\left(\frac{P_{r}}{P_{t}}\right). \end{array}$$
 Antenna-1 (horn) and Antenna-3 (AUT):
  (6) $$\begin{array}{c} G_{1\;\text{dB}}+G_{3\;\text{dB}}=20\;\text{lo}{\text{g}}_{10}\left(\frac{4\pi R}{\lambda }\right)+10\;\text{lo}{\text{g}}_{10}\left(\frac{P_{r}}{P_{t}}\right). \end{array}$$
 Antenna-2 (horn) and Antenna-3 (AUT):
  (7) $$\begin{array}{c} G_{2\;\text{dB}}+G_{3\;\text{dB}}=20\;\text{lo}{\text{g}}_{10}\left(\frac{4\pi R}{\lambda }\right)+10\;\text{lo}{\text{g}}_{10}\left(\frac{P_{r}}{P_{t}}\right), \end{array}$$

where *R* is the distance between the two antennas, *λ* is the transmit wave length, *P*_*r*_ is the received power level, and *P*_*t*_ is the transmitted power level.

The right hand side of (5), (6), and (3) can be calculated if the value of *R* and the ratio of received power/transmitted power are known. Thus, three unknown equations are produced with three unknown variables as follows:

(8) $$\begin{array}{c} G_{1\;\text{dB}}+G_{2\;\text{dB}}=X,\\ G_{1\;\text{dB}}+G_{3\;\text{dB}}=Y,\\ G_{2\;\text{dB}}+G_{3\;\text{dB}}=Z. \end{array}$$

The solution of (8) is easy and simple to calculate gain of three antennas

(9) $$\begin{array}{c} G_{3\;\text{dB}}\left(\text{AUT}\right)=\frac{Y-X+Z}{2}. \end{array}$$

**Figure 9:**
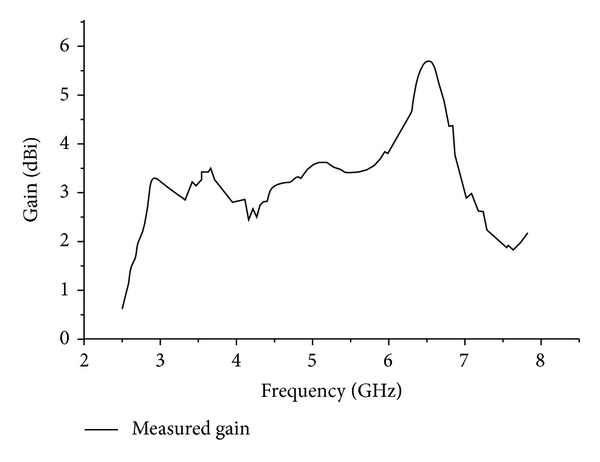
Measured gain of the proposed antenna.

Figure 9 shows the measured and simulated gains against the corresponding operating frequency bands. For the lower operating bands at 2.53–2.89 GHz the average gain is 2.43 dBi whereas for the band 3.47–3.91 GHz the gain is observed to be 2.67 dBi. The average gain for upper band at 6.28–7.83 GHz is achieved, 4.57 dBi, which in turn increases directivity of the designed antenna.

**Figure 10:**
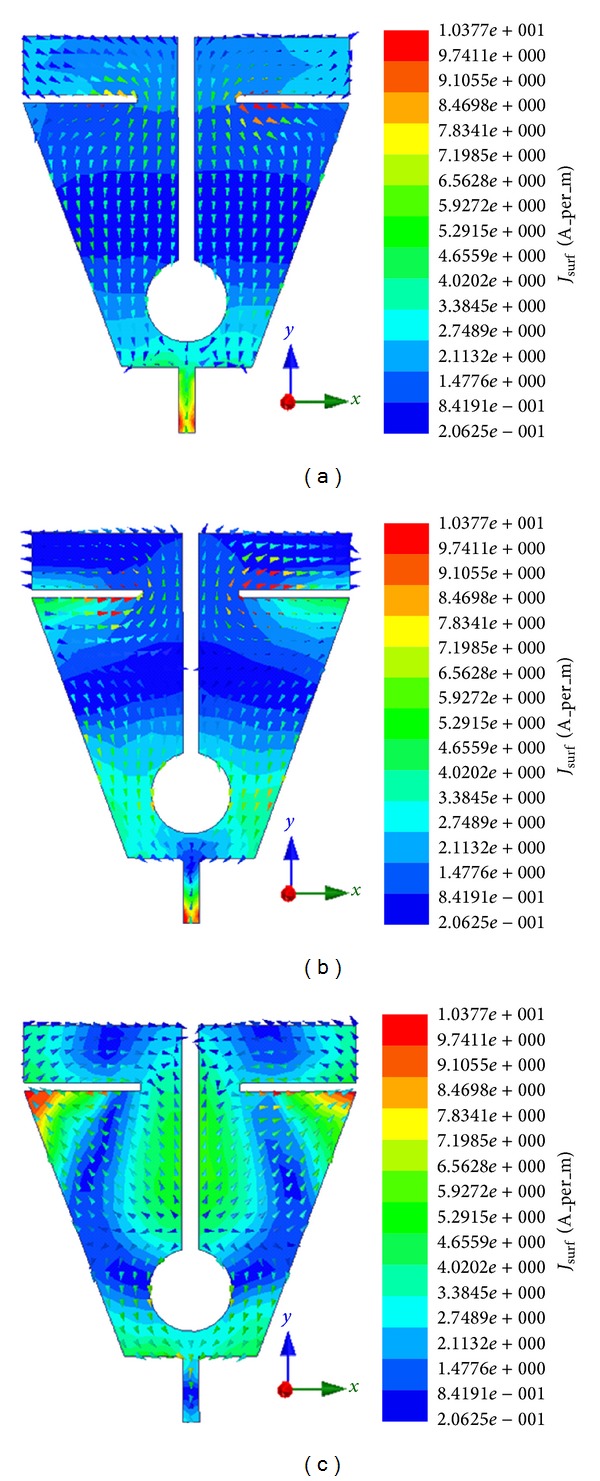
Illustration of surface current distribution of the proposed patch for (a) 2.66 GHz, (b) 3.65 GHz, and (c) 6.58 GHz.

**Figure 11:**
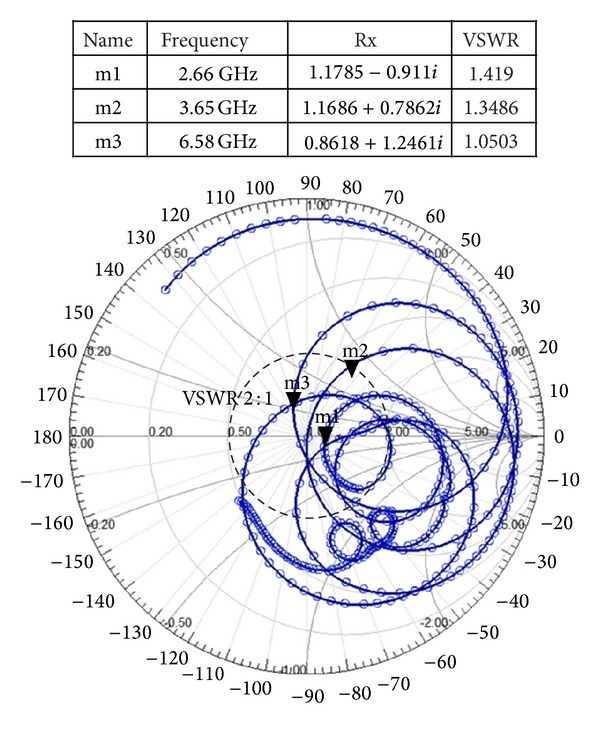
Smith chart of the proposed antenna.

Figure 10 illustrates the simulated surface current distribution of the radiating patch element of the proposed antenna at 2.66, 3.65, and 6.58 GHz resonant frequencies, respectively. It has been revealed through observation that the distribution of current is much stronger in upper band resonant frequencies than the lower band resonant frequencies which in turn validate gain. In a normalized scale, the voltage standing wave ratio (VSWR) and input impedance at operating frequency bands for the designed antenna are evaluated in Smith chart. Using normalization impedance of 50Ω, it is plotted on 2D complex reflection coefficient plane as shown in Figure 11. From the observation, it is found that three resonance frequencies of the antenna are inside the 2:1 VSWR circle. It is found that the input impedance curve has multiple twisted and overlapping circles at the center of the Smith chart which validate the multiband characteristics. Alongside, the tabular data are presented where *R*_*x*_ values are for the input impedance whereas the marked points *m*_1_, *m*_2_, and *m*_3_ indicate three resonant frequencies of the antenna.

Comparison between the proposed antenna and some existing antennas based on the applications and substrate material similarity is tabulated in Table 2. Nevertheless, the compared antennas are of similar material, but distinct resonant frequencies are observed due to the different radiating structures. Through reviewing the itemized performance characteristics, the reported antennas are larger in dimension or narrower in bandwidth or lower in gain compared to the proposed microstrip-fed patch antenna.

## 4. Conclusion

In this paper, a cost effective monopole antenna of 20 × 20 × 1.6 mm^3^ has been proposed and experimentally analyzed. The multiband capability of the antenna has been achieved with simple design characteristics by introducing different slots in the radiating patch without adding extra spare elements. The geometrical structure of the antenna has been synthesized and optimized by using the market available 3D electromagnetic simulator. Both the numerical analysis and measured results for VSWR, gain, and radiation patterns for the proposed antenna represent adequate performances with good agreement between them. The experimental results for the prototype antenna reveal the operating bandwidths of 360 MHz (2.53–2.89 GHz), 440 MHz (3.47–3.91 GHz), and 1550 MHz (6.28–7.83 GHz) with 3.62 dBi, 3.67 dBi, and 5.7 dBi gain, respectively. The comparison between the proposed and material/application specific antennas shows that the proposed antenna has significantly reduced dimension with better performance characteristics. Furthermore, the proposed antenna exhibits an almost steady radiation pattern with acceptable gain, which can satisfactorily cover the requirement offered by two WiMAX bands and C-band.

## Figures and Tables

**Table 1:** Design parameter for the proposed antenna geometry.

Parameter	Value (mm)
*W*	20
*W*_1_	9.5
*W*_2_	8
*W*_3_	0.5
*W*_*g*_	6
*W*_*m*_	1
*h*	1.6
*R*	2.5
*L*	20
*L*_1_	16
*L*_2_	3.5
*L*_*s*_	0.5
*L*_*g*_	4
*L*_3_	4
*L*_*m*_	13.5

**Table 2:** Performance comparison between the proposed antenna and some existing antenna.

Reference	Substrate material	Patch area (mm^2^)	Bandwidth (MHz)	Max gain (dBi)	Applications
[6]	FR4	25 × 25	300, 500, 700	2.15, 2.47, 4.13	WiMAX/WLAN
[24]	FR4	23 × 30	290, 290, 700	2.29, 0.9, 3.45	WiFi/WiMAX/C-band
[25]	FR4	40 × 50	180, 150, 170	1.7, 2.3, 4.1	Tri-band wireless
Proposed	FR4	20 × 20	360, 440, 1550	3.62, 3.67, 5.7	WiMAX/C-band
